# Simulation and Optimization of Connection-Strength Performance of Axial Extrusion Joint

**DOI:** 10.3390/ma15072433

**Published:** 2022-03-25

**Authors:** Jianguo Wu, Jingyu Zhai, Yangyang Yan, Hongwei Lin, Siquan Chen, Jianping Luo

**Affiliations:** 1 School of Mechanical Engineering, Dalian University of Technology, Dalian 116024, China; w1511291884@mail.dlut.edu.cn; 2 School of Mechanical Engineering, Weifang University of Science and Technology, Weifang 261000, China; yyysar@163.com; 3 Dalian C & L Technology Development Co., Ltd., Dalian 116000, China; hongwei_jialin@163.com (H.L.); m13478619391@163.com (S.C.); 4 AVIC Chengdu Aircraft Design & Research Institute, Chengdu 610000, China; lpgl159@163.com

**Keywords:** axial extrusion joint, experimental simulation of joint strength, joint-strength mechanism, grey relational analysis, back-propagation neural network

## Abstract

Axial extrusion-connection technology is one of the important connection technologies for hydraulic piping systems, with high sealing performance and mechanical strength. In this paper, the finite-element-modeling method is used to simulate the experimental process of the connection strength of the axial extrusion joint. The generation mechanism and calculation method of the connection strength are analyzed. To optimize the joint strength, orthogonal testing and grey correlation analysis are used to analyze the influencing factors of joint strength. The key factors affecting joint strength are obtained as the friction coefficient $\mu_{1}$, $\mu_{2}$ between joint components and the groove angle $\theta_{1}$ of the fittings body. The back-propagation (BP) neural-network algorithm is used to establish the connection-strength model of the joint and the genetic algorithm is used to optimize it. The optimal connection strength is 8.237 kN and the optimal combination of influencing factors is 0.2, 0.4 and 76.8°. Compared with the prediction results of the neural-network genetic algorithm, the relative error of the finite-element results is 3.9%, indicating that the method has high accuracy. The results show that the extrusion-based joining process offers significant advantages in the manufacture of high-strength titanium tubular joints.

## 1. Introduction

With the increase of the scale and complexity of aircraft, automobile and other products, the demand for new functions connecting mechanical components is also increasing. The connection based on plastic deformation has great potential in improving the accuracy, reliability and environmental safety of connection components [1]. Especially in hydraulic piping systems, tubular fittings are distributed in various parts of the hydraulic system to ensure the delivery of fluids from one location to another [2]. The connection technology between the pipeline fittings provides assurance of the safety and reliability of the whole piping system.

High-strength titanium-alloy (TA) tubes not only have good room temperature and high-temperature mechanical properties and corrosion resistance, but also have excellent cold- and hot-processing plasticity and formability [3,4]. It is suitable for high-pressure, lightweight hydraulic and fuel pipeline systems [5]. However, the traditional flaring joining technology cannot meet the joining and formation of titanium-alloy conduits, and axial extrusion-joining technology is considered to be one of the effective technical methods for the connection of conduits. The axial extrusion joint is a shape-closed joint based on plastic deformation [6], which has high-pressure resistance and mechanical strength, good self-locking performance and light weight. It is suitable for pipeline systems that do not need to be disassembled [7].

To date, many types of pipeline connection components have been studied. Yang et al. discussed the connection mechanism of an inner-diameter rolling joint by combining finite-element modeling and experimental verification [8]. Gies, S. et al. analytically estimated the internal pressure of a partially expanded pipe to completely fill the annular groove, carried out some studies on deformation-based pipeline connections and verified them experimentally [9]. Wang established the equivalent rigid model of a titanium-alloy pipeline connection and the simulated contact model under tensile load, and summarized the law of contact change on the sealing surface of the pipeline connection structure, so as to quantitatively analyze the sealing performance of the connection structure [10]. Alves et al. proposed a novel plastic-deformation process for joining two tube ends. The connection can be made at room temperature by internal mechanical locking and can ensure uniformity of the outer diameter of the two tubes to be connected [11]. Yan et al. established a multiscale model of the sealing area of pipeline joints with rough surfaces based on the measured data of the rough surfaces on the joints and obtained the sealing state and sealing performance of the joints through the simulation of the joint-tightening process [12]. Kang et al. established a smooth frictionless finite-element model of a shape-memory-alloy pipe joint and connected the pipe by using the function of the ABAQUS subroutine. The influence of joint-geometry parameters and expanding pressure on seal contact pressure is analyzed [13]. Zhou et al. used ANSYS to simulate and calculate the sealing properties of movable shape-memory-alloy pipe joints. The results show that the width of the sealing surface and the contact stress change with the structural parameters, which have a greater impact on the sealing [14]. Kyong-ho et al. studied the residual stress-conduction analysis and three-dimensional thermoelastic-plastic analysis of a welded-joint steel pipe by using the three-dimensional unsteady thermal analysis method and studied the mechanical properties of the welded joint [15]. Jeon et al. carried out a numerical simulation analysis of beam-sealed pipe joints and studied the effects of seal tilt angle and seal thickness on the sealing performance. A reasonable design of sealing tilt angle of 8.5° and sealing thickness of 0.45 mm was presented [16]. Prodan carried out a numerical analysis on the structural dimensions that affect the sealing performance of conical head-cone hole-type pipe joints [17]. The above pipeline-joining methods mainly focus on joint forming and sealing performance, but for aviation pipeline connection parts, attention should also be paid to joint strength.

In recent years, many achievements have been made in the connection strength of pipe fittings. Zhang et al. developed a model for spin forming and tensile testing of copper tubes and investigated the effect of forming parameters on the tensile strength of connected tubes and the connection mechanism [18]. Yamamoto et al. conducted joint-strength tests on shape-memory-alloy axial joints at different deformation rates and discussed the rate sensitivity of the joint strength [19]. Marré, M. et al. established an analytical model for the connection strength of aluminum-alloy tube-mold mating joints and used finite-element simulation and experiments to verify the model [20]. Yu et al. investigated a new method for plastic joining of thin-walled pipes through compression instability, where the geometry of the fold during forming determines the strength of the joint [21]. Henriksen et al. proposed a new method for a weldless connection of a pipe to a flange and evaluated the joining performance of the pipe to the flange after the connection and verified it experimentally [22]. Arvind, K. proposed a new method of joining two different-diameter steel pipes at both ends by plastic deformation and performed tensile and compression tests on the joints to verify the good joint strength [23]. Weddeling, C. used an electromagnetic forming–joining method to manufacture an aluminum-alloy joint consisting of a tube and mandrel and investigated the effect of groove structure on joint strength [24]. Zeng investigated the effect of two tube configurations, a triangular groove and rectangular groove, on the flow of titanium tubes. It was shown that the triangular groove-shaped tubing joints had better connection-strength performance [25].

There are few reports on axial extrusion joints and their connection strength needs to be further studied. In this paper, the simulation of the joint-strength experiment and the joint-strength-performance analysis are carried out based on an axial extrusion-joint model using ABAQUS. The joint-strength mechanism is discussed and the joint strength values are calculated. Through orthogonal experiments and grey correlation analysis, the order of the correlation degree of each factor to the connection strength is obtained and the key factors affecting the connection strength are acquired. A high-precision mathematical model of the connection strength of pipe joints is established by an optimized Latin hypercube-sampling method and BP neural-network algorithm. Its optimization is sought by using a genetic algorithm. The optimal joint strength and the best combination of influencing factors are obtained, which provide a reference for the structure and process design of axial extrusion joints.

## 2. Methods

### 2.1. Experimental Scheme of Joint Strength

**Figure 1:**
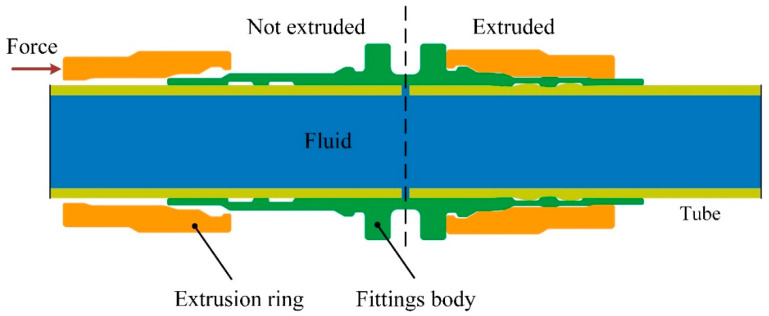
Axial extrusion-joint model.

The axial extrusion joint consists of tube, fittings body and extrusion ring, with a grooved structure in the middle of the fittings body. The extrusion ring is pushed axially along the body of the fitting using an extrusion tool. The extrusion ring squeezes the body of the joint and the tube so that the tube is embedded in the groove structure of the body of the joint, forming a mechanical connection and a metal seal. In this paper, an axial extrusion-joint model was designed based on the above principles and its two-dimensional model is shown in Figure 1. Two grooves are designed on the fittings body, and the mating surfaces of the extrusion ring and the fittings body are both designed as a three-stage stepped structure.

**Figure 2:**
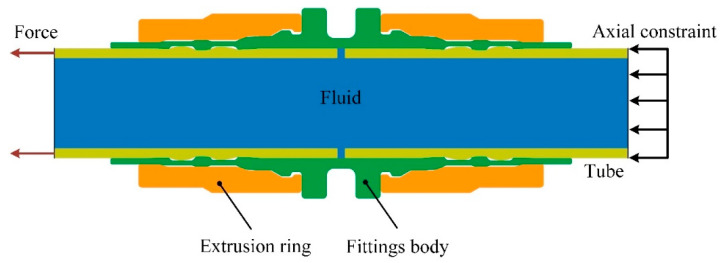
The principle of connection-strength experiment.

The principle of the connection-strength experiment of axial extrusion joint is shown in Figure 2. The axial extrusion joint is connected to the tensile-test machine. While maintaining the working pressure of the system internally, the titanium tube on the right side is fixed and the titanium tube on the left side is axially stretched until the connector leaks, pulls off or breaks. The maximum breaking force is the connection strength of the axial extrusion joint.

### 2.2. FE Modeling of Joint-Strength Experiment

**Figure 3:**
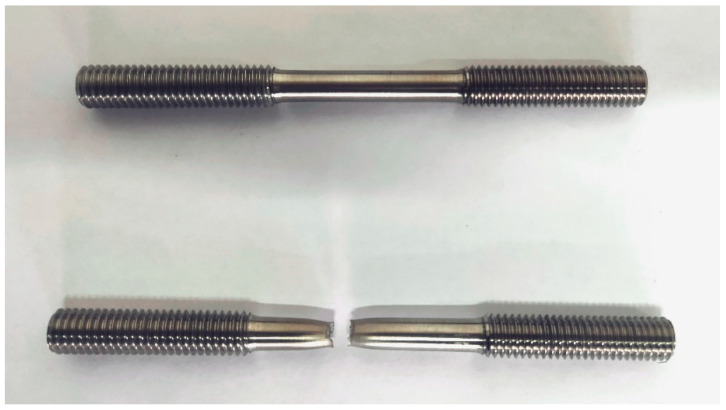
Tensile specimens before and after experiments.

**Figure 4:**
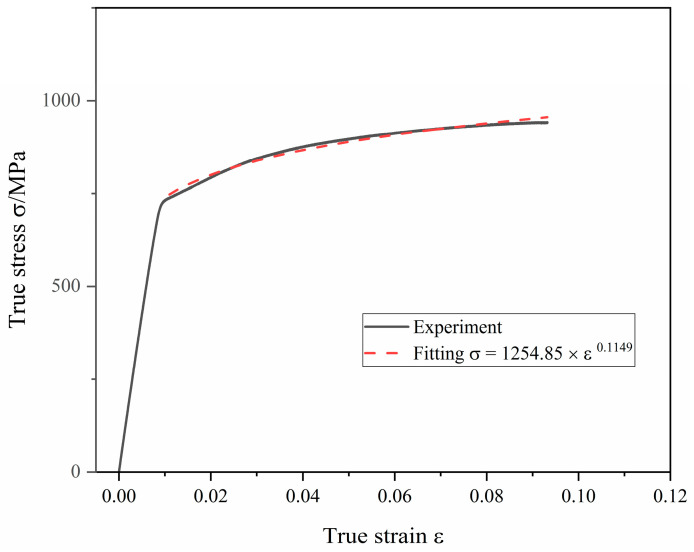
True stress–strain curve and hardening fitting of titanium tube.

The forming and drawing process of axial extrusion joint is axisymmetric, so a two-dimensional axisymmetric model was adopted. A longitudinal plane of the joint model was intercepted for simulation. When calculating the connection strength of the joint, in order to save calculation resources, the joint can be divided into two parts from the middle, intercepting only the left axially tensile part. The axial constraint of the right titanium tube can be equated to the fittings body. The components of the axial extrusion joint include the extrusion ring (Ti-6Al-4V), the fittings body (Ti-6Al-4V) and the titanium tube (Ti-3Al-2.5V). The deformation of the three components enters the plastic phase in the extrusion-forming process. According to the GB/T 228.1-2010 standard, the plastic zone data of the joint-component material were obtained through the uniaxial tensile test. Tensile specimens of extrusion-ring and fittings-body materials were processed and uniaxial tensile tests were carried out. The tensile specimens before and after the experiments are shown in Figure 3. The diameter of the titanium tube is 6 mm and its thickness is 0.5 mm. Uniaxial tensile test was performed on titanium-tube slices with full size of 180 mm. The true stress–strain curve and hardening fitting of titanium tube are shown in Figure 4. In order to obtain the large deformation data of plastic zone, the hardening model $\sigma =K\varepsilon^{n}$ was used to fit the true stress–strain curve of titanium tube. The regression value was 0.9837, indicating that the fitting of plastic zone curve has high precision. The mechanical properties of the joint component deduced from the uniaxial tensile test are listed in Table 1.

**Figure 5:**
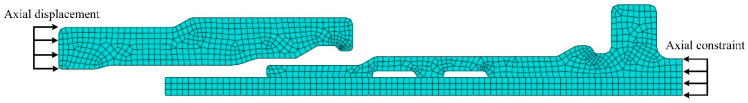
The extrusion-forming simulation of joint.

**Figure 6:**
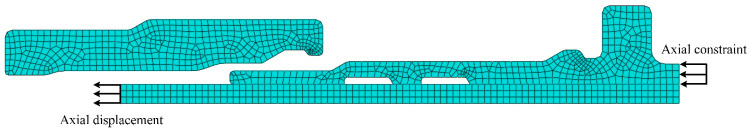
The simulation of tube pulling.

In view of the complex contact conditions in the forming and drawing process, the display-dynamics algorithm was used for analysis. The components of the axial extrusion joint all adopted the CAX4R linear solid element with reduced integral. In order to facilitate the contact calculation and avoid the distortion of the grid, the mesh size was set to 0.18 mm. In the forming and drawing process, the contact-constraint algorithm of each component adopted the dynamic-contact algorithm. The friction between the contact surfaces was calculated by the penalty-function model. The friction coefficient of the contact between the extrusion ring and the fittings body was set to 0.1 and the friction coefficient of the contact between the fittings body and the titanium tube was set to 0.2. The whole simulation process is divided into two steps: the first step is the joint-extrusion-forming process; the second step is the titanium-tube-pulling process, as shown in Figure 5 and Figure 6. In the first step, the initial boundary conditions of the joint included axial displacement constraints on the fittings body and the right side of the titanium tube. To simulate the extrusion process of the joint, an axial displacement load of amplitude 8 mm was applied to the left end face of the extrusion ring. In the second step, since it is difficult to simulate the drawing process of the titanium tube using force load, the problem was simplified to apply an axial displacement of 3 mm on the left side of the titanium tube, while removing the axial displacement constraint on the right side of the titanium tube.

## 3. Results and Discussion

### 3.1. Joint-Strength Mechanism

**Figure 7:**
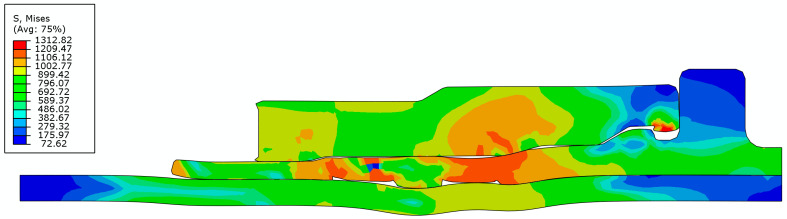
The von Mises stress distribution of axial extrusion joint after extrusion.

**Figure 8:**
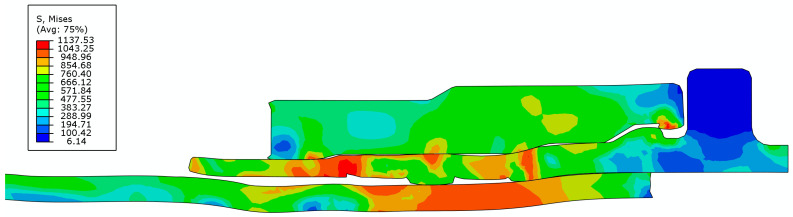
The von Mises stress distribution of axial extrusion joint after connection-strength experiment.

Figure 7 and Figure 8 show the von Mises stress distribution after the extrusion-forming and joint-strength experiments of the axial extrusion joint, respectively. As seen from the above figures, the initial contact condition is destroyed by the axial relative sliding between the titanium tube and the fittings body after the titanium tube is pulled. In the joint-strength experiment, the axial movement of the titanium tube requires overcoming the axial pull-off resistance of the titanium-tube material embedded in the groove of the fittings body. The titanium tube can be pulled off only after the titanium-tube material embedded in the groove of the fittings body is destroyed.

**Figure 9:**
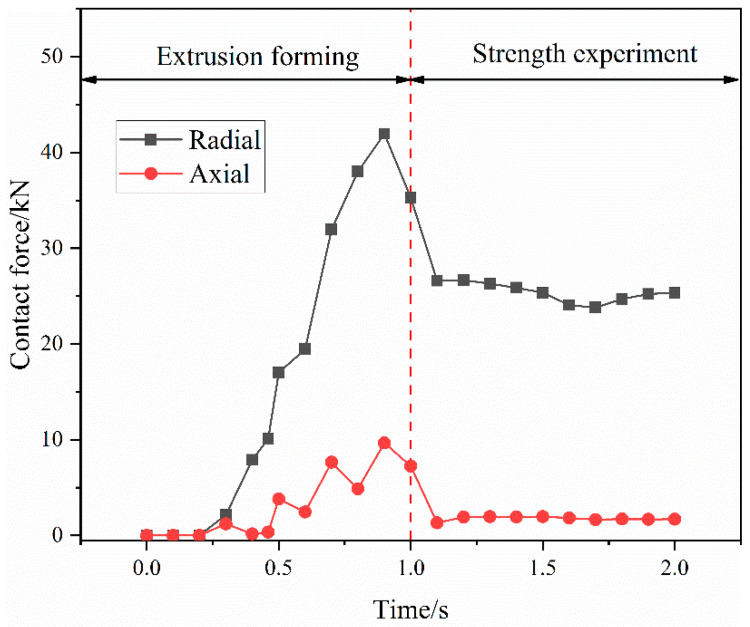
The contact-force variation curve between the extrusion ring and the fittings body.

Figure 9 illustrates the contact-force variation curve between the extrusion ring and the fittings body during the connection process. It can be seen that the contact force between the extrusion ring and the fittings body gradually increases during the extrusion-forming stage. When the time is 1 s, the axial extrusion joint is extruded. The radial contact force is 35.27 kN and the axial contact force is 7.25 kN. After the start of the joint strength experiment, the contact force between the extrusion ring and the joint body gradually decreases. After the contact between the titanium tube and the fittings body is broken, a constant residual contact force is maintained between the extrusion ring and the fittings body, which is 20.36 kN in the radial direction and 1.7 kN in the axial direction. There is no axial relative sliding between the extrusion ring and the fittings body, and they are in close contact.

**Figure 10:**
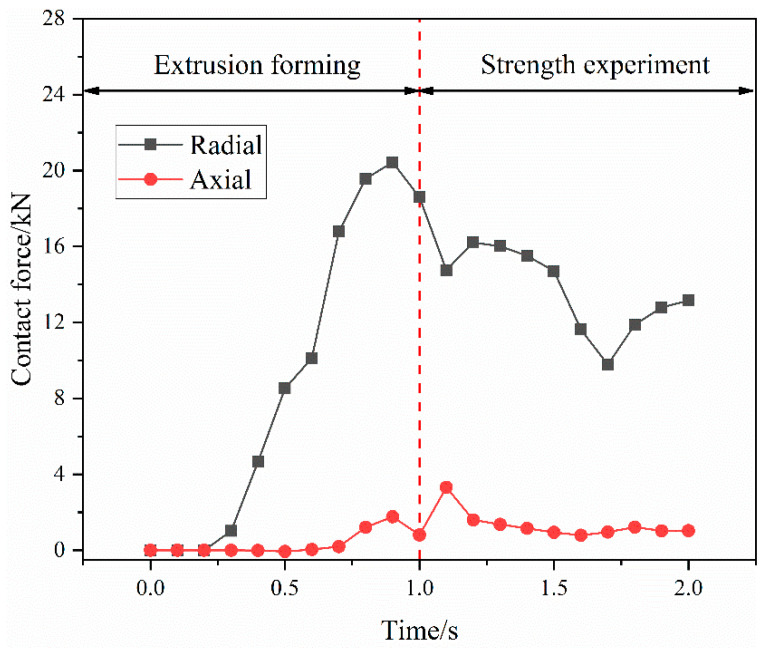
The contact-force variation curve between the fittings body and titanium tube.

Figure 10 illustrates the contact-force variation curve between the fittings body and titanium tube during the connection process. In the extrusion-forming stage, the contact force between the fittings body and the titanium tube increases gradually and the radial force is much larger than the axial force. At the end of the extrusion process, a constant residual contact force is maintained between the fittings body and the titanium tube, with a radial contact force of 18.58 kN and an axial contact force of 0.81 kN. The contact between the fittings body and the titanium tube is tight. After the connection-strength experiment, the contact state between the fittings body and the titanium tube is destroyed. The radial contact force drops sharply and the axial contact force shows a trend of increasing and then decreasing with time. The increased axial contact force between the fittings body and the titanium tube is caused by the pull-off resistance of the titanium-tube material embedded in the groove of the fittings body. Therefore, the connection strength of the joint is formed by both the radial contact force between the titanium tube and the fittings body and the axial pull-off resistance of the titanium-tube material embedded in the groove of the joint body, in which the radial contact force is the main part.

**Figure 11:**
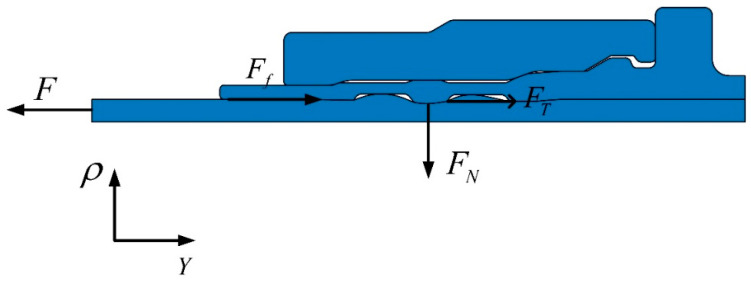
Force analysis on titanium tube during the pull-off process.

In order to calculate the value of connection strength, force analysis is carried out on the titanium tube during the pull-off process, as shown in Figure 11. During the pull-off process, the titanium tube is subjected to the pulling force F, frictional force $F_{f}$, radial contact force $F_{N}$ and axial pull-off resistance $F_{T}$. In the joint-strength experiment, the kinetic energy of material deformation is less than 5% of the internal energy and the whole system is quasi-static. That is, at any point in the process, the system is infinitely close to equilibrium. Thus, F can be obtained by Equation (1) in the axial direction.

(1) $$F=F_{f}+F_{T}=\mu_{2}F_{N}+F_{T}$$

**Figure 12:**
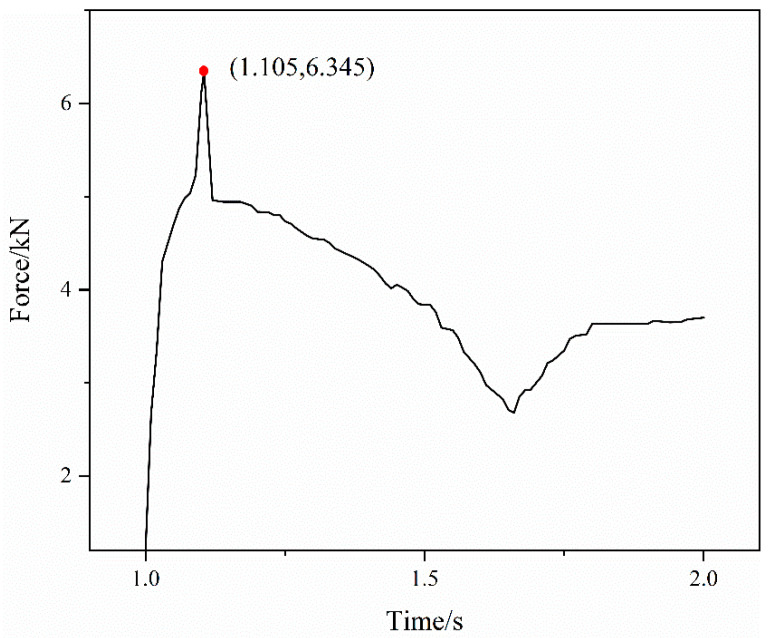
The variation curve of the pulling force on the titanium tube.

$\mu_{2}$ is the coefficient of friction between the fittings body and the titanium tube. The variation curve of the pulling force on the titanium tube can be calculated and is shown in Figure 12. The pulling force first increases sharply to a peak and then decreases slowly. The red point in Figure 12 represents the peak of the pulling force, which is the break point of the joint connection strength. The peak value is 6.345 kN. This is because when the pulling force reaches its peak, axial sliding occurs between the fittings body and the titanium tube and the close contact state is destroyed. Therefore, when the pulling force is greater than or equal to 6.345 kN, the connection state of the joint will be destroyed and the titanium tube will be pulled off. That is, the connection strength of the joint is 6.345 kN.

### 3.2. Experimental Design of Influencing Factors on Joint Strength

It can be seen from the above Equation (1) that the structural forming effect and the friction coefficient between the components have a great influence on the connection strength. The structural forming effect is mainly investigated in terms of the angle of the groove end of the fittings body and the extrusion-forming time. The friction coefficient between the components includes the friction coefficient $\mu_{1}$ between the extrusion ring and fittings body and the friction coefficient $\mu_{2}$ between the titanium tube and the fittings body.

**Figure 13:**
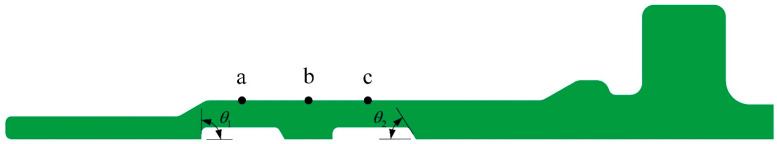
The structure of the fittings body.

**Figure 14:**
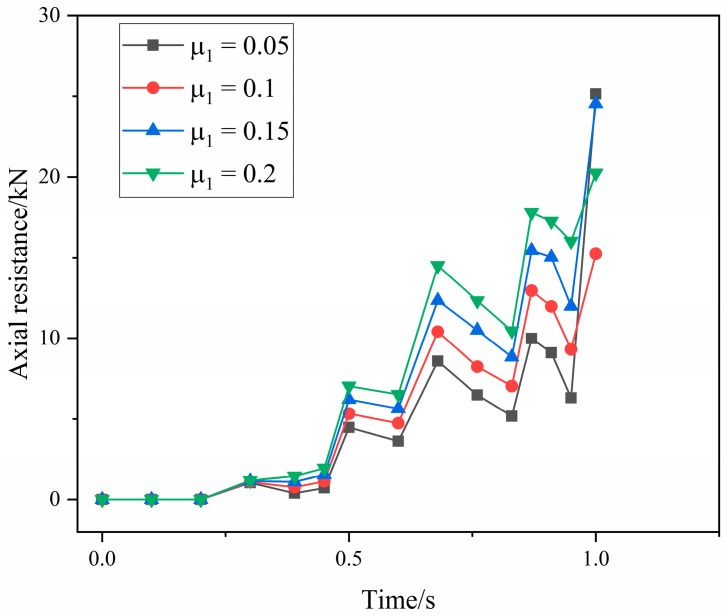
The variation of axial resistance between the extrusion ring and fittings body under different friction coefficients.

**Figure 15:**
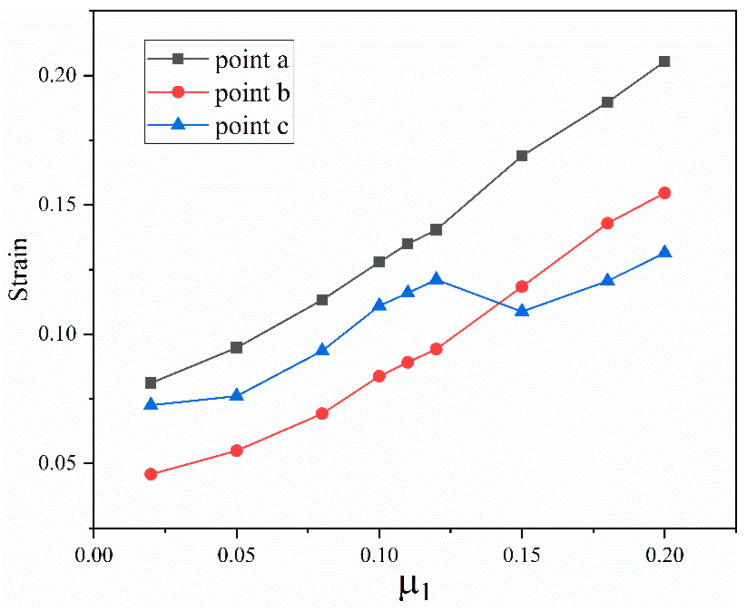
Variation of strain after forming with different friction coefficients at points a, b and c.

The extrusion-forming process of the axial extrusion joint is a cold-forming process. The heat generated by friction and the temperature change of the joint components is small. The heat generated by friction during extrusion has little influence on the joint-forming effect. The friction coefficient affects the deformation distribution of the component material by changing the resistance of the material during the forming process. Figure 13 shows the structure of the fittings body. In order to more clearly study the change of material-strain distribution under different friction coefficients, three points, a, b and c, on the upper surface of the fittings body are selected. Figure 14 shows the variation of axial resistance between the extrusion ring and the fittings body during the forming process under different friction coefficients. It can be seen that with the increase in the friction coefficient, the axial resistance clearly increases during the forming process. Figure 15 shows the strain changes after forming at points a, b and c under different friction coefficients. The strain at the three points shows an obvious upward trend, so it can be seen that the change in the friction coefficient between surfaces has a certain influence on the forming effect of the pipe joint. If the friction coefficient is too large, the resistance of the material becomes larger and the material flow is difficult, which leads to the forming difficulty of the joint and the uneven distribution of the material deformation. If the friction coefficient is too small, the material deformation will be small and the joint extrusion forming is not sufficient. According to actual production experience, the range of $\mu_{1}$ and $\mu_{2}$ is 0.05–0.2 and 0.2–0.4, respectively, and five horizontal values are taken respectively in the range. The five level values of $\mu_{1}$ are 0.05, 0.08, 0.1, 0.15 and 0.2. The five level values of $\mu_{2}$ are 0.2, 0.25, 0.3, 0.35 and 0.4.

The extrusion time of the joint may affect the plastic deformation and flow effect of material, and thus affect the joint-forming effect and joint strength. Combined with the actual production, the simulation time of joint extrusion forming is less than 1 s. Five forming times are given in this paper, which are 0.1 s, 0.25 s, 0.5 s, 0.75 s and 1 s, respectively.

As shown in Figure 13, the fittings body has two grooves of the same structure. The left and right ends of the groove angle are $\theta_{1}$ and $\theta_{2}$, respectively. $\theta_{1}$ and $\theta_{2}$ can affect the plastic deformation and flow of the titanium tube to a certain extent, and thus affect the final forming quality of pipe joint. Too small an angle will lead to incomplete material flow and affect the tensile force of axial material. $\theta_{1}$ and $\theta_{2}$ range from 50° to 90°. Five typical values are selected in this paper, which are 50°, 60°, 70°, 80° and 90°, respectively.

According to the influencing factors of connection strength and their value range, the test factor level is shown in Table 2. Taking the connection strength as the target value, the orthogonal table of $L_{25}\left(5^{5}\right)$ was selected to carry out the orthogonal test. The test results are shown in Table 3.

### 3.3. Grey Correlation Analysis

Grey correlation refers to the uncertain correlation between things or between system factors and main behavior factors. The purpose of grey correlation analysis is to find a quantitative method that can measure the degree of correlation between various factors [26,27]. In this paper, the grey correlation theory is applied to the analysis of influencing factors of joint-connection strength, which can quickly determine the correlation between each parameter and the connection strength and obtain the key parameters that affect the connection strength.

The general calculation steps for grey correlation analysis are as follows. There is a sequence $\;X=\left\{\begin{array}{c} x_{i}|i\in M,M=\left\{1,2,\ldots ,m\right\},m\geq 2,x_{i}=\left(x_{i}\left(1\right),x_{i}\left(2\right),\ldots ,x_{i}\left(k\right)\right)\\ x_{i}\left(k\right)\in x_{i},k\in K,K=\left\{1,2,\ldots ,n\right\},n\geq 2 \end{array}\right\}$, where k is the index and m is the sequence number. Equation (2) is used to standardize the original data.

(2) $$f\left(x_{i}\left(k\right)\right)=\frac{x_{i}\left(k\right)-\underset{i\in M}{min}x_{i}\left(k\right)}{\underset{i\in M}{max}x_{i}\left(k\right)-\underset{i\in M}{min}x_{i}\left(k\right)}$$

$x_{0}\in X,x_{0}=\left\{x_{0}\left(k\right)|k=1,2,\ldots ,n\right\}$ is selected as the reference sequence; $x_{i}\in X$ is the comparison sequence; $x_{0}\left(k\right)$ and $x_{i}\left(k\right)$ are the data of $x_{0}$ and $x_{i}$ at the k index, respectively. According to Equation (3), $\xi_{0i}\left(k\right)$ is defined as the grey correlation coefficient of $x_{i}$ to $x_{0}$ at point k under a certain environment on X.

(3) $$\xi_{0i}\left(k\right)=\frac{\underset{i\in M}{min}\underset{k\in K}{min}\left|x_{0}\left(k\right)-x_{i}\left(k\right)\right|+\rho \underset{i\in M}{max}\underset{k\in K}{max}\left|x_{0}\left(k\right)-x_{i}\left(k\right)\right|}{\left|x_{0}\left(k\right)-x_{i}\left(k\right)\right|+\rho \underset{i\in M}{max}\underset{k\in K}{max}\left|x_{0}\left(k\right)-x_{i}\left(k\right)\right|}$$

where ρ is the resolution factor, generally taken as ρ equal to 0.5; $\left|x_{0}\left(k\right)-x_{i}\left(k\right)\right|$ is the distance; $\underset{i\in M}{min}\underset{k\in K}{min}\left|x_{0}\left(k\right)-x_{i}\left(k\right)\right|$ and $\underset{i\in M}{max}\underset{k\in K}{max}\left|x_{0}\left(k\right)-x_{i}\left(k\right)\right|$ are the minimum and maximum difference between the two levels, respectively.

The measurement of the correlation between two systems or two factors is called the correlation degree. According to Equation (4), the non-negative real number $r\left(x_{0},x_{i}\right)$ is defined as the gray correlation degree of $x_{i}$ to $x_{0}$.

(4) $$r\left(x_{0},x_{i}\right)=\frac{1}{n}\overset{n}{\underset{k=1}{\sum }}\xi_{0i}\left(k\right)$$

The data in Table 3 were taken into Equations (2)–(4) and calculated. The correlations of $\mu_{1}$, $\mu_{2}$, forming time, $\theta_{1}$ and $\theta_{2}$ with the connection strength of the joint are 0.6503, 0.7534, 0.5967, 0.6587 and 0.6055, respectively, which shows that the correlation of $\mu_{2}$ is the largest and the correlation of forming time and $\theta_{2}$ is smaller than that of $\mu_{1}$ and $\theta_{1}$. The forming time and $\theta_{2}$ are considered to have a smaller effect on the joint strength, so the forming time is subsequently taken to be 1 s and $\theta_{2}$ to be 60°. The key factors affecting the joint strength are $\mu_{1}$, $\mu_{2}$ and $\theta_{1}$.

### 3.4. Optimization of Influencing Factors of Joint Strength

**Figure 16:**
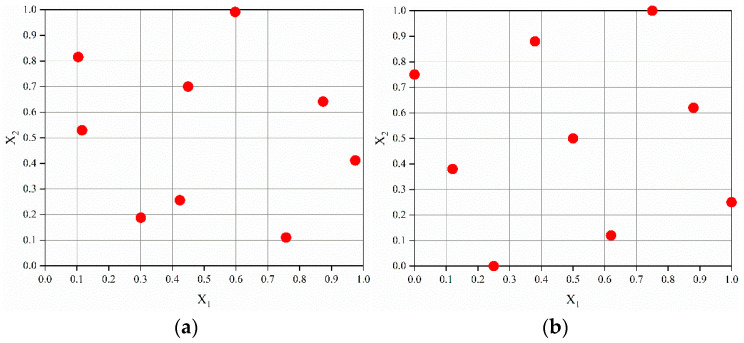
Random and optimal Latin hypercube-sampling trial-point distributions: (**a**) random Latin hypercube sampling, (**b**) optimal Latin hypercube sampling.

**Figure 17:**
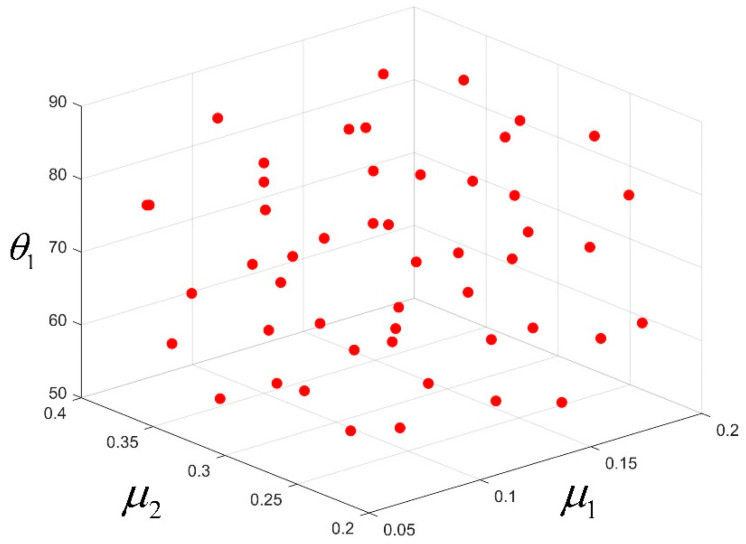
Sample of connection-strength influencing factors.

The parameter setting in the orthogonal test above is too absolute. In order to improve the accuracy of subsequent fitting and optimization, The Latin hypercube-sampling method is adopted to determine the input parameters. The Latin hypercube design is loose in its division of level values and the number of trials can be artificially controlled. However, there may still be a less-than-even distribution of test points, and the possibility of losing some areas of the design space increases as the number of levels increases. The optimal Latin hypercube design improves the inhomogeneity of the random Latin hypercube design and makes all the test points evenly distributed in the design space as far as possible [28], which has very good space filling and balance. Figure 16 demonstrates the two-dimensional spatial trial-point distribution for the random and optimal Latin hypercube-sampling designs. It can be seen that optimal Latin hypercube sampling produces a more uniform distribution of trial points. Based on the optimal Latin hypercube-sampling method, $\mu_{1}$, $\mu_{2}$ and $\theta_{1}$ are sampled within their corresponding ranges to obtain 50 groups of samples, as shown in Figure 17.

**Figure 18:**
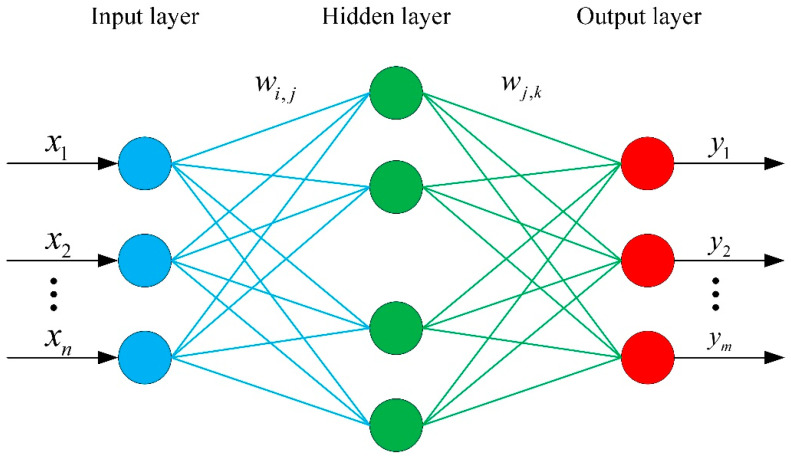
BP neural-network framework.

BP (back-propagation) neural network is a kind of neural network trained according to the error back-propagation algorithm, which has a strong nonlinear-mapping capability and can realize the prediction of the output data results given the input data [29,30]. A neural-network structure is generally composed of three parts: input layer, hidden layer and output layer. The three-layer BP neural-network structure can satisfy the result that the output is close to the actual expected value under the given input value, so as to achieve the prediction target. The general framework is shown in Figure 18. Based on the modeling purpose, the neurons in the input layer are determined as three: $\mu_{1}$, $\mu_{2}$ and $\theta_{1}$. The neurons in the output layer are determined as one: connection strength. The number of neurons in the hidden layer is usually determined according to Equation (5).

(5) $$l=\sqrt{m+n}+a$$

where l is the number of neurons in the hidden layer; m is the number of nodes in the input layer; n is the number of nodes in the output layer. a is an integer from 1 to 10. The range of hidden neurons obtained by substituting data is [3,13]. The number of hidden neurons obtained by trial algorithm is 8.

**Figure 19:**
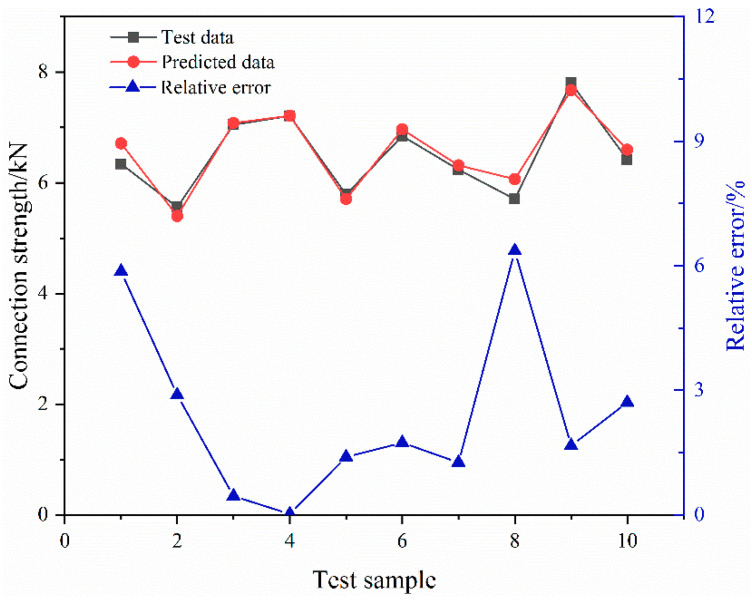
Comparison of predicted output and expected output.

The above 50 groups of samples are used as neural-network training data. The optimal Latin hypercube-sampling method is used to sample another 10 groups of samples as test data. The BP neural-network model with the structure of 3-8-1 is built in MATLAB, and the training function is selected as Levenberg–Marquardt. The learning rate is set to 0.01. The corresponding mean square error is 0.03702 and the regression value $R^{2}$ is 0.956. It is generally believed that the smaller the value of the mean square error, the closer $R^{2}$ is to 1, the better the training effect. Figure 19 shows the distribution of predicted and expected outputs. The black points show the connection-strength data from the finite-element simulation. The red points are the data simulated by the neural-network model. The blue points are the relative error. The maximum relative error is 6.36%, and the minimum relative error is 0.021%. It shows that the data predicted by the neural-network model are consistent with the data obtained from the finite-element simulation, and the error between the two is small. The neural-network model has high precision.

**Figure 20:**
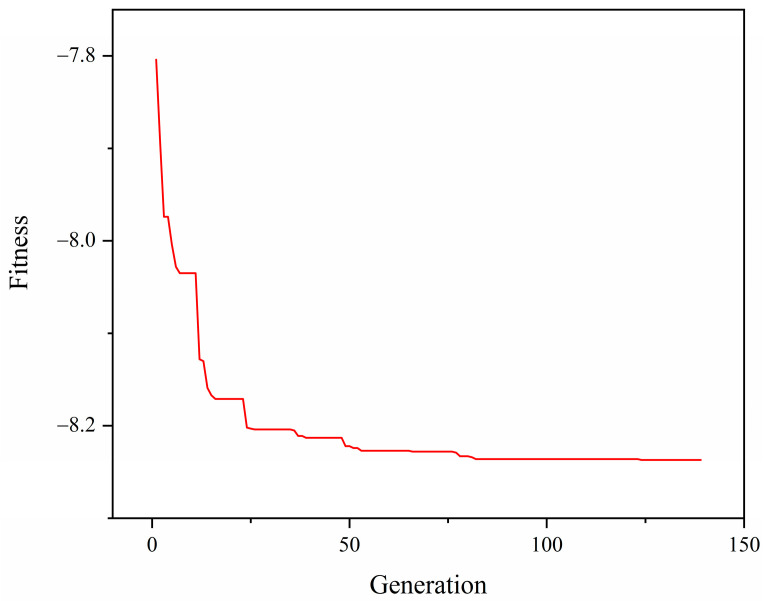
The variation curve of fitness with iterations of population evolutionary generations.

The genetic algorithm is an effective method for solving complex nonlinear optimization problems using simulated biological evolutionary processes [31]. The genetic algorithm consists of five parts: genetic code, individual fitness, selection stage, crossover operation and mutation operation. The genetic algorithm is usually used to find the minimum value of the model. The maximum value of the joint-connection strength is required in this paper, so the fitness is the negative value of the joint connection strength. The binary code with strong searching ability is adopted. The population size is 50, the crossover probability is 20%, the mutation probability is 20% and the iterative convergence accuracy is set to 1 × 10^−6^. Figure 20 illustrates the variation curve of fitness with iterations of population evolutionary generations. After 139 iterations, the maximum joint-connection strength is 8.237 kN; corresponding variables are (0.2, 0.4, 76.8), i.e., $\mu_{1}$ is 0.2, $\mu_{2}$ is 0.4, $\theta_{1}$ is 76.8°, as shown in Figure 20. The optimal key influence factors obtained above are input into the finite-element model and the strength of the pipe joint connection is obtained as 7.928 kN. Compared with the neural-network prediction results, the relative error between the two is 3.9%. It indicates that the method has high prediction accuracy and achieves the purpose of optimizing the key influence factors.

## 4. Conclusions

In this paper, the connection-strength performance of the axial extrusion joint is studied in terms of finite-element modeling and simulation, joint-strength mechanism and strength-influencing factor analysis. The connection-strength experimental process, connection-strength calculation method, correlation analysis and optimization of connection-strength-influencing factors of the joint are investigated. The following conclusions can be derived:

(1) Based on the joint-strength experimental scheme of the axial extrusion joint, finite-element modeling of the joint was performed using Abaqus to simulate the extrusion-forming and titanium-tube pull-off process of the joint. The forces between the fittings were analyzed and the mechanism of joint-strength generation was analyzed. It is shown that the connection strength of the joint is formed by both the radial contact force between the titanium tube and the fittings body and the axial pull-off resistance of the titanium-tube material embedded in the groove of the joint body. The calculated connection strength of the axial extrusion joint is 6.345 kN.
(2) The factors influencing the joint strength include the angle of the notch ends of the joint body, the extrusion time and the intercomponent friction coefficient. The orthogonal-level test was conducted according to the number and level of the test factors with the joint strength as the target value. Gray correlation analysis was performed on the orthogonal test results to obtain the correlation ranking of each factor on the joint strength. The key factors affecting the joint strength are obtained as the friction coefficient $\mu_{1}$ between the extrusion ring and the joint body; the friction coefficient $\mu_{2}$ between the titanium tube and the joint body; and the angle $\theta_{1}$ of the left end of the groove.
(3) In order to optimize the connection strength of the axial extrusion joint, the optimized Latin hypercube-sampling method was used to sample the three key influencing factors. The BP neural-network algorithm was used to establish a mathematical model of pipe joint strength. The mean square error of the model is 0.03702 and the regression value is 0.956, indicating that the neural network has high prediction accuracy. The genetic algorithm was used to optimize the neural-network model. The optimal connection strength is 8.237 kN and the optimal combination of influencing factors is 0.2, 0.4 and 76.8°. Compared with the prediction results of the neural-network genetic algorithm, the relative error of the finite element results is 3.9%, indicating that the method has high accuracy.

## Figures and Tables

**Table 1:** Material mechanical properties for the joint component.

Materials	Elastic Moduli (GPa)	Yield Strength (MPa)	Tensile Strength (MPa)	Elongation (%)
Extrusion ring	122.11	1053	1143	15.7
Fittings body	116.54	950	1010	6.9
Tube	103	730	864	12

**Table 2:** Joint connection-strength-test factor level.

Level	$\mu_{1}$	$\mu_{2}$	Time/s	$\theta_{1}$	$\theta_{2}$
Level 1	0.05	0.2	0.1	50	50
Level 2	0.08	0.25	0.25	60	60
Level 3	0.1	0.3	0.5	70	70
Level 4	0.15	0.35	0.75	80	80
Level 5	0.2	0.4	1	90	90

**Table 3:** Results of orthogonal test.

Test Number	$\mu_{1}$	$\mu_{2}$	Time/s	$\theta_{1}$	$\theta_{2}$	F/kN
1	0.05	0.2	0.1	50	50	4.9117
2	0.05	0.25	0.25	60	60	5.3649
3	0.05	0.3	0.5	70	70	6.6833
4	0.05	0.35	0.75	80	80	7.0905
5	0.05	0.4	1	90	90	6.9565
6	0.08	0.2	0.25	70	80	5.0455
7	0.08	0.25	0.5	80	90	6.4287
8	0.08	0.3	0.75	90	50	6.0699
9	0.08	0.35	1	50	60	6.5218
10	0.08	0.4	0.1	60	70	7.0614
11	0.1	0.2	0.5	90	60	6.3271
12	0.1	0.25	0.75	50	70	5.5689
13	0.1	0.3	1	60	80	6.1745
14	0.1	0.35	0.1	70	90	6.7317
15	0.1	0.4	0.25	80	50	7.2050
16	0.15	0.2	0.75	60	90	5.3123
17	0.15	0.25	1	70	50	6.3642
18	0.15	0.3	0.1	80	60	7.1325
19	0.15	0.35	0.25	90	70	7.3789
20	0.15	0.4	0.5	50	80	7.2457
21	0.2	0.2	1	80	70	6.2761
22	0.2	0.25	0.1	90	80	6.6211
23	0.2	0.3	0.25	50	90	6.3385
24	0.2	0.35	0.5	60	50	7.3973
25	0.2	0.4	0.75	70	60	7.9805

## Data Availability

The data presented in this study are available on request from the corresponding author.
